# The evolving landscape around genome editing in agriculture: Many countries have exempted or move to exempt forms of genome editing from GMO regulation of crop plants

**DOI:** 10.15252/embr.202050680

**Published:** 2020-05-19

**Authors:** Sarah M Schmidt, Melinda Belisle, Wolf B Frommer

**Affiliations:** ^1^ Heinrich Heine University Düsseldorf Germany; ^2^ Bill & Melinda Gates Foundation Seattle WA USA

**Keywords:** Plant Biology, S&S: Economics & Business

## Abstract

The EU and New Zealand are the only legislations where genome‐edited plants are considered and regulated as GMOs while many other countries move to exempt genome‐edited crops.

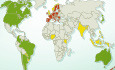

Genome editing is revolutionizing plant science and its applications in agriculture. In its simplest form, it can generate specific genetic variants that are indistinguishable from naturally evolved variants. The legislation and regulation of genome‐edited plants in many countries is similarly evolving rapidly to adapt to the new technologies. Here, we summarize and provide an assessment of the current status of this rapidly evolving regulatory landscape, with a focus on recent policy developments in Europe and the global South.

The legislation and regulation of genome‐edited plants in many countries is similarly evolving rapidly to adapt to the new technologies.

Genome editing by site‐directed nucleases (SDNs) such as TALEN or Cas9 is a versatile tool that generates variations in the recipient genome at specific target sites. SDNs produce a sequence‐specific DNA break that is repaired by the plant's natural DNA repair mechanisms; as the repair is inherently imperfect, it results in target‐site variants. Depending on the type of approach, we can distinguish between three types of alterations. SDN‐1 introduces base‐pair changes or small insertions/deletions without addition of foreign DNA. The exact change cannot be predetermined and is quasi random at the target site. SDN‐2 uses a small DNA template to generate a specific change by homologous recombination. SDN‐3 inserts larger DNA elements of foreign origin using a similar approach as SDN‐2 (ref. 1); the introduction of larger pieces of DNA is typically considered as transgenic. Many countries have now adapted their biosafety legislation based on this classification of SDN‐induced variants (Fig 1).

**Figure 1:**
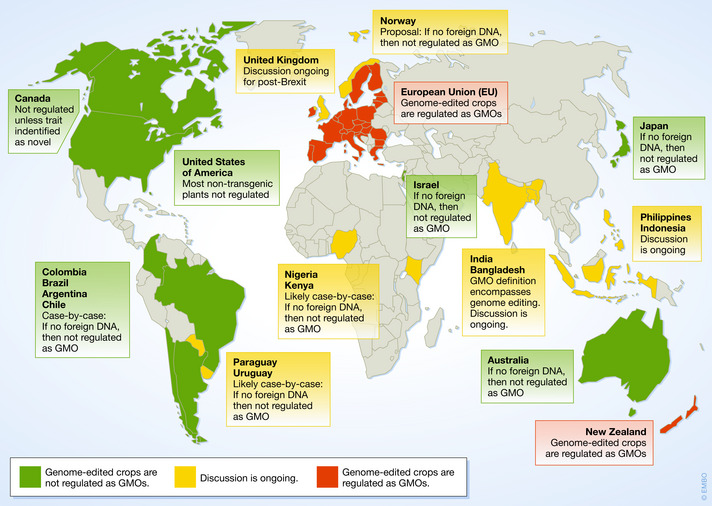
**Current state of genome‐editing legislation**.

## Developments in Europe

In Europe, the EU Court of Justice (ECJ) ruled in 2018 that all genome‐edited organisms must be categorized as genetically modified organisms (GMO) and are therefore subject to significant regulatory burdens under the EU GMO Directive (Box 1). Although chemical and radiation mutagenesis techniques are exempted from the Directive, the ECJ ruled that genome editing could not be exempted 1 because these techniques were developed after the GMO directive was written. Furthermore, the ECJ explained that an exemption for genome‐edited products would not correspond with the spirit of the law, whereas products developed using conventional mutagenesis are exempted because of a history of safe use. Other than the EU, only New Zealand regulates genome editing under its GM biosafety rules, also via a court decision.

The ECJ ruling bewildered scientists and had a negative impact on agricultural innovation. Big agricultural companies moved their advanced breeding programs out of Europe. Only 8% of CRISPR patents in agriculture come from Europe, while 60% originate from China and 26% from the USA 2. Critics called for the new EU Commission to amend the GMO legislation, and, in October 2019, the Council of the European Union requested a study from the Commission to determine the status of genome‐editing techniques to ensure compliance with the Directive (Box 1). The Commission contends that the study, scheduled to be completed by April 2021, will examine the question of enforcement as well as current and future applications of genome editing, a risk assessment framework developed by the European Food Safety Authority (EFSA) and, lastly, ethical and societal implications (Box 1). Although the request for the study does not specifically request an update of the existing legislation, it does say that the Commission should “submit a proposal, if appropriate in view of the outcomes of the study”.

Other than the EU, only New Zealand regulates genome editing under its GM biosafety rules, also via a court decision.

In addition to an assessment of the potential risks and ethical considerations, we believe that EFSA should also consider the socio‐economic implications of any regulatory decisions: consequences for researchers and farmers locked out of technological advances, potential trade disruptions, and the impacts on food security in developing countries. A wholescale rejection of innovation in agriculture also limits options to achieve sustainability and climate‐change targets and diminishes European exceptionalism. As there is little quantitative analysis available in the published literature, EFSA has to solicit a range of perspectives to deliver a robust assessment. Stakeholder consultations are ongoing, and the EU commission discussed a draft survey with a body of stakeholders including NGOs, industry, and scientific organizations, such as the European Sustainable Agriculture through Genome Editing (EU‐SAGE) network, which represents 131 European plant science institutes (Box 1).

It is unlikely that the situation can remain as is, since it places an undue burden on member states to enforce a technically unenforceable law since SDN‐1 and SDN‐2 products are indistinguishable from natural variants or variants generated by mutagenesis. However, any attempt to make changes to the GMO Directive through the legislative process risks unravelling the entire European GMO regulatory system. Some of the options available to EFSA include either tracing each product entering the EU market back to its originally source, or defining a timeframe by which a product could be considered to have a history of safe use, and thereby qualifies as an exempted form of mutagenesis. It remains to be seen which approach will be taken but we posit that the least disruptive scenario is most likely.

Separately, the governments of Switzerland, Norway, and the UK are considering new laws to facilitate approval of products from genome editing. Norway is currently affiliated to the EU's authorization procedures for GMO under directive 2001/18/EC, but the Norwegian Biotechnology Advisory Board proposed in December 2018 to exempt SDN‐1 events and to implement an expedited assessment for SDN‐2 events (Box 1).

## India as an example for the importance of deregulation

India recently requested public comments to inform their decisions on future policies on genome editing (Box 1). In January 2020, India released a draft document on genome‐edited organisms: “Regulatory Framework and Guidelines for Risk Assessment”. The document proposes a tiered approach to group products based on risk, with single or a few base‐pair edits identified as low risk—“Group 1”, and insertion of large or foreign DNA as higher risk—“Group 3”. The draft deals with genome‐edited plants, animals, and human cells in one document, which is rare among biosafety rules, and frequently conflates the risks associated with each. For instance, there is heavy reference to off‐target effects. While these may be a valid concern for medical products, the frequency of off‐target mutations using CRISPR‐Cas is low in plant cells 3, 4, 5 and off‐target mutations can easily be removed by crosses. Also, there is no differentiation between the health risks of off‐target mutations in somatic cells for human therapeutics versus plant cells where deleterious effects are eliminated by crossing and pose no ethical issues.

It is unlikely that the situation can remain as is, since it places an undue burden on member states to enforce a technically unenforceable law…

Additionally, any market authorization will require review by no less than three agencies: the Institutional Biosafety Committee, the Ministry of Agriculture & Farmers’ Welfare, and the Food Safety and Standards Authority of India. If India does not alter the planned legislation, it places itself somewhere in between the complete deregulation of SDN‐1 and SDN‐2 in Latin America and the United States and regulation of all genome‐edited plants as GMO in the EU.

It remains to be seen how difficult the introduction of SDN‐1 plants to the Indian market will actually be. The data requirements India requests in the draft, even for organisms deemed low risk, are considerably more than in other countries. Although chemically mutagenized plants can be immediately released for field testing, genome‐edited plants, according to the proposal, would require a compositional assessment, a full suite of molecular, phenotypic, and agronomic equivalence measurements, and, potentially, an environmental safety assessment. Together, this would add months or years of data collection to submit an SDN‐1 type edited product. Since agricultural products are of prime importance for food security, a simpler path to bring new plant varieties to farmers, especially those who are most vulnerable, seems highly important.

## The status in other parts of the world

In 2015, Argentina was the first country to formally declare that crops will not be regulated under biosafety legislation if the plant products do not contain foreign DNA (resolution no. 173/2015). Chile (normative resolution 2017), Brazil (normative resolution no.16/2018), and Colombia (resolution no. 29299/2018) soon followed. The respective biosafety authorities decide on a case‐by‐case basis and will only regulate a new plant product as a GMO if it contains a “novel combination of genetic material” 6. Paraguay and Uruguay declared their intention to adopt the same regulatory approach.

The United States has been deciding on a case‐by‐case basis whether to allow planting of SDN‐1 plants in the field since 2014. In 2018, the US department of Agriculture (USDA) decided not to impose regulation on new breeding technologies comprising genome editing (Box 1). Most plants produced by SDN‐1 or SDN‐2 events are not subject to regulation by USDA once the CRISPR gene has been crossed out. The US biosafety legislation is instead triggered by the presence of potential risk factors, such as the presence of T‐DNA from the plant pathogen *Agrobacterium tumefaciens* or toxicity. For example, blight‐resistant rice generated by TALENs was deregulated and can be planted in the field without a permit (Box 1). In Canada, the regulatory trigger is novelty, that is a lack of an existing history of safe use. For example, if natural variants have been used in the context of breeding pathogen resistance, respective edited plants would not be subject to biosafety regulation 7.

New Zealand's GMO legislation is currently applied to all genome‐editing events. In Australia, on the other hand, the Office of the Gene Technology Regulator (OGTR) does not regulate SDN‐1 type genome‐editing applications (edits without templates) since October 8, 2019 (Box 1).

Japan does not regulate plant varieties in a different manner than conventionally bred varieties, if they do not contain new DNA (SDN‐1 and SDN‐2). Indonesia and other countries in Southeast Asia are currently in the process of deciding whether to exempt crops produced by SDN‐1 or 2 from GMO legislation. Although the GMO definition in biosafety legislation of Bangladesh encompasses genome‐edited products, the government is discussing whether or not to regulate genome‐edited plants.

Although China has most publications and patents for gene‐editing applications in agriculture 8, they surprisingly have yet to establish a regulatory framework for evaluating genome‐edited plant products. This might reflect the fragmented Chinese biosafety legislation, which mostly comprises administrative measures and regulatory documents, but no specific law dedicated to biosafety. After He Jiankui announced in November 2018 that he had genetically modified human embryos via gene editing, regulation on human gene editing was tightened through yet another administrative regulation (Administrative Regulation on Human Genetic Resources in May 2019). However, the incident might have intensified concerns about genome‐editing technology in general with crops as collateral damage. Abdicating global leadership on agricultural genome‐editing policy is a missed opportunity for China given their influential position as a major trading partner for many countries and their leadership in research and development for agricultural applications.

The data requirements India request in the draft, even for organisms deemed low risk, are considerably more than in other countries.

South Africa currently treats genome‐edited crops as GMO, although a discussion about a policy amendment is ongoing. In Kenya and Nigeria, the National Biosafety Authorities are in the process of drafting guidelines for regulating genome‐editing technologies.

Israel does not regulate SDN‐1‐ and SDN‐2‐derived plant products if no foreign DNA is present (Box 1).

In summary, the list of countries with enabling legislation for genome‐edited crops is growing and many countries in Asia and Africa are discussing to not regulate genome‐edited crops as GMOs. Only in the EU and New Zealand, genome‐edited crops are placed under existing GMO biosafety by court ruling although it seems that the EU is currently revisiting this position. China is conspicuously absent from the policy discussion despite its many products in the pipeline.

## Benefits of deregulation

The public and civil organizations have been highly critical of GM plants mainly because of their association with herbicides and pesticides, a misguided view that these are an “unnatural” combination of organisms and a notion that these benefit mostly international companies. The burgeoning global policy landscape holds the potential to address at least two of these criticisms. First, SDN‐1 and SDN‐2 products are not a combination of organisms but could also have been generated by mutagenesis and/or conventional breeding. Second, a lower regulatory burden for genome‐edited products means a cheaper and faster path to market, which assures that small and medium‐sized enterprises and academic research institutes can afford to clear regulatory hurdles. If there is no need to generate revenue to pay for costly regulatory approvals, researchers can diversify the agricultural products and pursue humanitarian goals that do not generate profits. Early reports from Argentina indicate that their lighter approach to genome‐editing regulation has led to just this scenario: an increased percentage of product applications from public sector developers and a wider array of products submitted for regulatory review.

If there is no need to generate revenue to pay for costly regulatory approvals, researchers can diversify the agricultural products and pursue humanitarian goals that do not generate profits.

It will be interesting to follow the developments in Europe, India, China, and other countries that now develop or modify their policies. The increasing deregulation and the rapidly evolving regulatory landscape could mean that transgene‐free crops, for example, the recently developed broad‐spectrum blight‐resistant rice lines 9, 10, could finally be made available to those who need them most.

## Conflict of interest

WBF and SMS are using genome editing to generate robust broad‐spectrum resistance against rice blight in the framework of a strictly humanitarian project (http://www.healthycrops.org).

**Box 1: Policy documents relevant to genome editing**

*Confédération paysanne and Others v Premier ministre and Ministre de l'agriculture, de l'agroalimentaire et de la forêt*. (2018).

Council of the European Union. 12781/19. RP/IC/vm. LIFE.2.B. EN.Council of the European Union. Brussels, 24 October 2019. https://data.consilium.europa.eu/doc/document/ST-12781-2019-INIT/en/pdf.

EC study on new genomic techniques. https://ec.europa.eu/food/plant/gmo/modern_biotech/new-genomic-techniques.

Stakeholders’ consultation. https://ec.europa.eu/food/plant/gmo/modern_biotech/stakeholder-consultation_en.

Norwegian Biotechnology Advisory Board. Proposal for relaxation of European regulations for deliberate release of genetically modified organisms (GMO). http://www.bioteknologiradet.no/filarkiv/2019/01/Proposal-for-relaxation-of-GMO-regulations-with-annexes.pdf.

DBT invites comments on “Draft document on Genome Edited Organisms: Regulatory Framework and Guidelines for Risk Assessment” | Department of Biotechnology. http://dbtindia.gov.in/latest-announcement/dbt-invites-comments-%E2%80%9Cdraft-document-genome-edited-organisms-regulatory.

Mohanty, S. World Needs to Keep a Close Watch on Agriculture While Battling COVID‐19 Pandemic. *Sam's Agriculture and Food Security Blog agriculture* https://globalfoodsystems.org/world-needs-to-keep-a-close-watch-on-agriculture-while-battling-covid-19-pandemic/ (2020).

Secretary Perdue Issues USDA Statement on Plant Breeding Innovation. https://www.usda.gov/media/press-releases/2018/03/28/secretary-perdue-issues-usda-statement-plant-breeding-innovation.

https://www.aphis.usda.gov/biotechnology/downloads/reg_loi/aphis_resp_isu_ting_rice.pdf.

Health, A. G. D. of. 2019 Amendments to the Regulations

https://www.fas.usda.gov/data/israel-agricultural-biotechnology-annual-2.
